# Biofabrication of a Low Modulus Bioelectroprobe for Neurons to Grow Into

**DOI:** 10.3390/ma14164718

**Published:** 2021-08-21

**Authors:** Zhiyan Hao, Sen Wang, Kun Zhang, Jiajia Zhou, Dichen Li, Jiankang He, Lin Gao, Ling Wang

**Affiliations:** 1 State Key Laboratory for Manufacturing System Engineering, Xi’an Jiaotong University, Xi’an 710054, China; tonyhao@stu.xjtu.edu.cn (Z.H.); seeker@stu.xjtu.edu.cn (S.W.); zhoujiajia955@stu.xjtu.edu.cn (J.Z.); dcli@mail.xjtu.edu.cn (D.L.); jiankanghe@mail.xjtu.edu.cn (J.H.); gaolin2013@xjtu.edu.cn (L.G.); 2 School of Mechanical Engineering, Xi’an Jiaotong University, Xi’an 710054, China; 3 Department of Pharmacology, School of Pharmacy, Fourth Military Medical University, Xi’an 710032, China; b1074446046@stu.xjtu.edu.cn

**Keywords:** neural electrode, conductive biomaterial, polyaniline, glial response

## Abstract

Implantable nerve electrodes, as a bridge between the brain and external devices, have been widely used in areas such as brain function exploration, neurological disease treatment and human–computer interaction. However, the mechanical properties mismatch between the electrode material and the brain tissue seriously affects the stability of electrode signal acquisition and the effectiveness of long-term service in vivo. In this study, a modified neuroelectrode was developed with conductive biomaterials. The electrode has good biocompatibility and a gradient microstructure suitable for cell growth. Compared with metal electrodes, bioelectrodes not only greatly reduced the elastic modulus (<10 kpa) but also increased the conductivity of the electrode by 200 times. Through acute electrophysiological analysis and a 12-week chronic in vivo experiment, the bioelectrode clearly recorded the rat’s brain electrical signals, effectively avoided the generation of glial scars and induced neurons to move closer to the electrode. The new conductive biomaterial electrodes developed in this research make long-term implantation of cortical nerve electrodes possible.

## 1. Introduction

In recent years, cortical nerve electrodes, as a tool for studying neuroscience and understanding brain function, have received more and more attention due to their wide applications in biomedicine/rehabilitation (such as regulating the growth of neurons, using as the brain–computer interface, repairing the nervous system, etc.) [1,2,3,4,5,6,7]. Implantable nerve electrodes can mainly be divided into the following categories: the first one is a metal micro-wire electrode, which is made of gold, platinum, iridium, tungsten and other metals or metal alloys, and its diameter is generally less than 100 μm. The outer surface of the metal micro-wire electrode is covered with a layer of insulating materials such as polytetrafluoroethylene, teflon and polyimide. The end of the electrode forms a bare flat or conical tip by grinding, cutting and chemical corrosion, which is used for recording the nerve electrical signals [8]. Some researchers combined multiple micro-wire electrodes to form a metal micro-wire electrode array to improve the spatial resolution of the recording of the signal [9]. The second one is the Utah electrode, which was a needle micro-electrode array designed and manufactured by the University of Utah through microelectronics technology [10]. The needle length of the Utah electrode generally ranges from 35 to 75 μm. This electrode was generally used in the superficial part of nerve tissue or peripheral superficial nervous system due to the detection depth of this electrode being shallow. Another kind of electrode is the Michigan electrode, which is a rod-shaped electrode array made by Wise of the University of Michigan [11] by spraying conductive metal on the silicon substrate and etching conductive points at the end of the electrode. Some researchers use flexible polymers as substrates and packaging materials to make more flexible electrodes to reduce the impact and damage to the nerve tissue caused by the electrodes. However, the lead line of the Michigan electrode is thin, which is generally used to detect and record the electrical signals of nerve tissue.

It was still a major challenge for implantable nerve electrodes to record nerves signals stably for a long time after implantation. Electrode failure hinders the wide application of cortical nerve electrode technology in human clinical practice. On the one hand, the mechanical damage of the electrode is the main factor affecting the useful effect of the electrode. The nerve tissue can be scratched or squeezed after the electrode is implanted, and the electrode can put pressure on the tissue surrounding the electrode. Furthermore, the micro-movement between the electrode and brain tissue can continue to aggravate the mechanical damage to never tissue along with the use of the electrode. The smaller the difference of Young’s modulus between the nerve electrode and the brain tissue, the smaller mechanical damage caused by the electrode micro-movement to the brain tissue (1 KPa) [12]. On the other hand, the rejection of natural tissues caused the destruction of the interface between the electrode and tissue, which include the acute tissue damage caused by the implantation of the electrodes in the cortex [13,14]. Finally, the glial scar formed in the brain would reduce the accuracy of the probe over time. Astrocytes and microglia were the two main types of glial cells involved in the reaction of neural tissue. Microglia were activated after the electrode was implanted in the brain, the wound healing mechanism was activated through the soluble factors secreted by microglia, and the astrocytes were activated by the released inflammatory cytokines. The macrophages that can swallow the implanted foreign bodies were gathered around the activated microglia [15,16]. This acute inflammatory response may interrupt the recording of the electrode, but it usually subsides within a few weeks after the electrode is implanted in the brain. Then a longer-term chronic tissue response can be followed, which was characterized by the formation of an encapsulation layer mainly composed of astrocytes around the probe. The glial layer insulates the implant from adjacent nerve cells to increase the impedance of the electrode. This interruption may lead to the degradation of the quality of the signal and ultimately limit the function of the electrode.

Various methods for improving the electrode-cell interface and preventing the proliferation of glial cells around the electrode were studied. One way is to reduce the size of the electrode. Some studies showed that, compared with traditional silicon probes, subcellular-sized probes could significantly reduce the formation of glial scars and maintain recording quality for a long time [17,18]. For example, Edward et al. used the method of chemical, meteorological deposition to modify the multi-walled carbon nanotubes on the purchased tungsten wire microelectrodes, which effectively improved the electrical performance of the electrodes and the ability to collect nerve signals. The diameter of the electrode recording site is only about 10 μm [19]. Takashi D et al. used a carbon fiber microelectrode with a diameter of only 8 μm, which can effectively reduce tissue damage during electrode implantation [20]. The second way is the improvement of electrode materials. More flexible materials were selected to make the electrodes to reduce the mechanical damage caused by the mechanical mismatch between the nerve electrode and the adjacent tissue layer. There are many ways to make electrodes flexible. Firstly, flexible materials can be used to make electrode substrates. For example, C.H. Chen et al. used SU-8 glue as a substrate to prepare graphene photoelectrodes, which can be bent at will (90°) [21]. It is also possible to wrap a layer of flexible material around the electrode to increase the overall flexibility of the electrode. For example, Mohammad Reza Abidian et al. wrapped a layer of sodium alginate hydrogel around the silicon nerve electrode to reduce the tissue immune response of the electrode [22]. The third way is to modify the electrode to enhance the biocompatibility and conductivity of the electrode. Some researchers have reported that combining conventional nerve electrodes with anti-inflammatory drugs (such as dexamethasone (DEX, Flavopiridol) or nerve cell adhesion molecules) to change conventional nerve electrodes can prevent gliosis [23,24]. In addition, by coating the recording electrode with conductive materials such as carbon nanotubes and conductive polymers, it is also possible to reduce the electrode impedance for better bonding with nerve tissue. These interventions enhance the adhesion of nerve cells to the electrode to achieve higher recording effects by reducing the inflammatory response induced by the electrode or improve the electrode use time by increasing the electrode conductivity to improve the signal-to-noise ratio.

The ideal nerve electrode should have good biocompatibility and electrochemical stability. This kind of electrode can avoid scar wrapping, has good charge conductivity and realizes long-term electrical stimulation of nerve tissue [25]. We designed and manufactured a bio-conductive hydrogel electrode with good biocompatibility, stability and conductivity. Adjusting the mechanical properties of bio-conductive gel can make its modulus closer to natural tissue. The conductive gel layer can form a flexible buffer layer between the metal electrode (~200 GPa) and the natural brain tissue (~1 KPa), which reduces the mechanical damage of the electrode to the brain tissue.

## 2. Materials and Methods

### 2.1. Manufacturing of Biological Brain Electrodes

#### 2.1.1. Design of the Electrode

**Figure 1:**
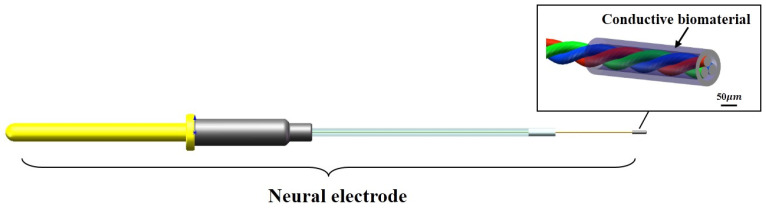
Low modulus bioelectrode design.

In view of the problems of glial scar caused by the existing implanted electrode, which leads to the failure of the electrode, the design of the electrode in this study was mainly optimized from the following two aspects. The first is to minimize the gap of modulus between the electrode and the brain tissue (1 KPa) on the premise of ensuring the mechanical stability of the electrode itself to reduce the mechanical damage to the brain tissue caused by the contact stress and electrode micro-movement. The second is that the gel layer coated around the electrode site could affect the conductivity of the electrode to the electrical signal. Therefore, while ensuring the good biocompatibility and stability of the gel material, it is also necessary to enhance its electrical conductivity to meet the demand for electrode charge injection (Figure 1).

#### 2.1.2. Preparation of the Bio-Conductive Material

The bio-conductive material of the bio-coating of the electrode was composed of the biological material and the conductive polymer. The preparation method of bio-conductive materials was as follows: sodium alginate powder (9005-38-3, Sigma, Burlington, MA, USA) and gelatin powder (9000-70-8, Sigma, Burlington, MA, USA) were dissolved in double distilled water, which was stirred with a magnetic stirrer in a water bath at 40 °C in order to prepare sodium alginate solution with a concentration of 2 wt.% and gelatin solution with a concentration of 10 wt.%. The gelatin solution and transglutaminase (TG enzyme, 80146-85-6, BoMei, North York, ON, Canada) solution were sterilized by using a filter (33 mm, SLGP033 RB, Millipore, Burlington, MA, USA) with a pore size of 0.22 μm. TG enzyme can promote the crosslinking of gelatin at room temperature. The sodium alginate solution and polyaniline powder (5612442, MACKLIN, Gateshead, UK) were sterilized by using a high-pressure steam sterilizer (DGL-50B, LICHEN, Loughborough, UK). In order to study the effect of the proportion of polyaniline in the mixture on the properties of the materials, bio-conductive gel with 0%, 3%, 5%, 7% and 9% polyaniline were prepared, respectively (0% means no polyaniline were added). First, the conductive polyaniline powder was slowly added to the gelatin solution in the 40 °C water bath, and then the mixed solution was stirred with a magnetic stirrer for 30 min and treated with an ultrasonic cleaner for 120 min. After that, the same volume of sodium alginate solution as gelatin solution was slowly added to the mixed solution, and then this solution was stirred with a magnetic stirrer for 30 min and treated with an ultrasonic cleaner for 120 min. Finally, 0.8%TG enzyme was added to form a bio-conductive gel solution with different concentrations of polyaniline −1 wt.% of alginate −10 wt.% of gelatin.

#### 2.1.3. Manufacturing Method of the Electrode

The implantable biological brain electrode was mainly composed of copper output interface, positioning sleeve, conductive core wire and biological conductive material. The conductive core wire was made of two nickel-chromium alloy wires with a diameter of 30 μm through the magnetic suspension winding method, and the conductive core wire was connected to the copper output interface through soldering technology. The copper output interface, the positioning sleeve and the conductive core wire were encapsulated together with a rubber packaging tube and polyethylene glycol. The nerve electrodes were immersed in 75% ethanol solution for 10 min and then washed three times with sterile phosphate-buffered saline (PBS). A dipping method was used to deposit the biological conductive gel coating, and the number of leaching times of the sample was monitored with an optical microscope (SZM03590, AOMEI, Chengdu, China) to control the thickness of the bio-conductive gel coating. The samples were processed by cooling, gradient vacuum drying from −40 to 20 °C using the freeze dryer (Pilot2-4M, Boyikang, Beijing, China) for 48 h, and soaking in 0.1 mol/L CaCl_2_ solution in sequence, the bio-conductive material forms a porous bio-conductive layer with a diameter about 100 μm on the tip of the electrode, and then the sample was stored in a sterilized dish at room temperature.

### 2.2. Performance Testing of the Conductive Materials

#### 2.2.1. Test Method of the Mechanical Properties

A multifunctional mechanical testing machine (ETM 103A) was used to measure the compressive modulus of the bio-conductive materials. A 5 × 5 × 5 mm^3^ standard bio-conductive material sample was prepared with a silicone mold to measure the compression modulus of this material. The force–displacement curve of the material was obtained by controlling the displacement (2 mm/min) through a mechanical sensor (with an accuracy of 0.01 N) with a measuring range of 20 N, and then the compressive elastic modulus of the material can be calculated. Scanning electron microscopy (SEM, Hitachi su-8010, Tokyo, Japan) was used to observe the pore size on the surface of the bio-conductive gel layer and the distribution of the polyaniline. The scanning voltage was set as 5 kV, and the surface morphology of the bio-conductive materials was observed when the concentration of polyaniline was 0%, 3%, 5%, 7% and 9%.

#### 2.2.2. Test Method of the Electrical Performance

The conductivity of the bio-conductive materials can directly affect the charge conductivity of the materials, so the different concentrations of conductive polymer added to the materials can affect the conductivity of the bio-conductive materials. The conductive samples (*n* = 4) containing different concentrations of polyaniline with a size of 6 × 6 × 5 mm^3^ prepared in this experiment were placed between two parallel copper electrodes. A multimeter was used to measure the DC resistance of the sample, and the conductivity was calculated.

Cyclic voltammetry and electrochemical impedance spectroscopy (EIS) were used to analyze the electrochemical performance of the nerve implants. The experimental device was a three-electrode system, which was composed of an experimental electrode immersed in PBS, a counter electrode and a reference electrode. Ag/Ag Cl was used as a reference electrode, Pt was used as a counter electrode and the experimental electrodes were metal electrodes and biological electrodes (*n* = 3). A 100 mV sine wave was used to measure the impedance spectrum (EIS) of the electrode, and the frequency range ranges from 1 Hz to 100 kHz.

#### 2.2.3. Biocompatibility In Vitro

U87 cells (TCHu138, National Collection of Authenticated Cell Cultures) were used to test the biocompatibility of the prepared materials. The cells were cultured in an H-DMEM medium (Hyclone, Logan, UT, USA) containing 10% fetal bovine serum (Sigma, Burlington, MA, USA) and 1% penicillin-streptomycin (Sigma, Burlington, MA, USA) in an incubator with a temperature of 37 °C and 5% CO_2_. Bio-conductive films were prepared on the bottom of the 48 well plate, and 8 × 10^4^ U87 cells were seeded in each well. The experiment was divided into 5 groups. The experimental group was the bio-conductive gel with 3%, 5%, 7% and 9% polyaniline, respectively, and the control group was the bio-gel without polyaniline. Cell adhesion and synaptic growth were observed at 4 h, 1 day and 4 days of culture.

### 2.3. Detection of Biocompatibility of Electrode In Vivo

Adult Sprague-Dawley rats (SD rat) weighing about 250 g were selected for the electrode implantation experiment. The experimental group was 1 mm bio-conductive material-coated electrodes, and the control group was 1 mm metal electrodes. The rats were anesthetized with chloral hydrate (diluted with 5% normal saline and injected in the abdominal cavity at a ratio of 2 mL/300 g), and the heads of the rats were shaved and fixed on a stereotaxic device. After the head of the rat was disinfected and the skin with a length of about 2.5 cm was cut along the midline to expose the surface of the skull. A portable rechargeable bone drill was used to open a hole about 1 mm in diameter at 2.3 mm in front of the anterior fontanel and 2.5 mm in the left and right sides of the midline. Then the broken bone was washed out with PBS, and the bio-electrode and metal electrode were vertically implanted into the left and right foramen, respectively. The implanted depth of the electrode in the brain was 1 mm, and the end of the electrode was aligned with the surface of the cortex. The implanted area of the electrode was in the motor cortex of rats.

The animals (*n* = 4) of two groups were sacrificed at 4 weeks, 8 weeks and 12 weeks. First, the animals were deeply anesthetized with excessive chloral hydrate. After the animals were anesthetized, the heart of the animal was perfused with PBS at room temperature, and then the heart was perfused with 4% paraformaldehyde (4 °C) to fix the tissue. After the brain tissue was removed, the biological electrode and metal electrode were removed under the stereomicroscope. The whole brain was stored in 4% paraformaldehyde for 48 h, and then the brain was transferred to 75%, 85%, 95% and 100% ethanol solution, respectively, for gradient dehydration. After the brain was transparent by xylene, it was embedded in paraffin. The brain tissue was sliced in a direction parallel to the axis of the electrode. The thickness of the slice was 8 μm. One slice was taken as a specimen for every five slices, and the slice depth of the samples was 1500 μm. Hematoxylin-eosin staining (HE staining) and immunofluorescence staining (DAPI/MAP2/GFAP/Iba-1) were used to analyze the effect of implanted electrodes on the brain tissue.

### 2.4. Test of Electrode Acute Electrophysiological Recording

**Figure 2:**
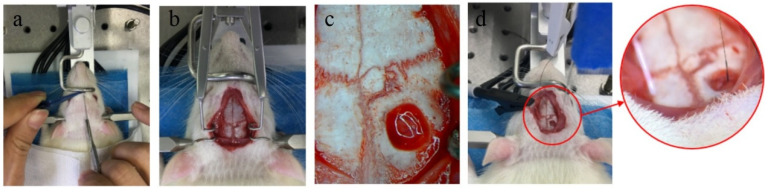
Acute electrophysiological recording process. (**a**) Strip the rat scalp; (**b**) expose skull; (**c**) skull drilling; (**d**) implant electrode.

The nerve signals were collected immediately after the implantable biological brain electrode was implanted in the brain, which were used as the data of acute recording. Nerve signals were collected by the BL-420F biological function experimental system. The bio-electrode was inserted vertically into the rat motor cortex with a depth of 0.5 mm by a stereotaxic device (Figure 2). The acquisition frequency of all channels was set to 40 k Hz, and the band-pass filter frequency ranged from 300 to 5000 Hz. The ground electrode and the reference electrode were connected with the skull of the rat through the screws fixed on the skull, and the ground electrode was connected with the ground electrode of the acquisition system.

### 2.5. Statistical Analysis

The experimental data were expressed as mean ± standard error (Mean ± SD). The difference between the data of two groups was tested by a two-tailed T-Test; the difference between the data of multiple groups was analyzed by one-way and two-way ANOVA, where *p* < 0.05 indicates significant statistical differences in the data (* *p* < 0.05, ** *p* < 0.01).

## 3. Results

### 3.1. Mechanical Properties

**Figure 3:**
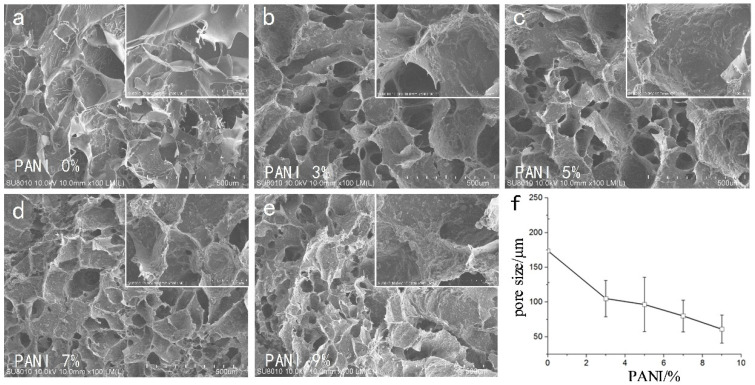
Porous structure of the bio conductive materials with different polyaniline concentrations. (**a**–**e**). SEM images of bio conductive materials at 0%/3%/5%/7%/9% polyaniline concentration. (**f**) Pore change curve of bio conductive materials with different polyaniline concentrations.

We used CAD software to manually measure the size of the pores in each group (*n* = 4, 50 pores of each SEM photo). The SEM results (Figure 3) showed that the pore size of the material was 175 ± 48 μm without adding conductive polymer. With the increase in polyaniline concentration, the porosity of the material decreased gradually. From the 500 SEM images, it could be seen that the surface roughness of micropores increased with the increase in polyaniline concentration (Ra 0.181 ± 0.066 μm ~0.621 ± 0.118 μm).

**Figure 4:**
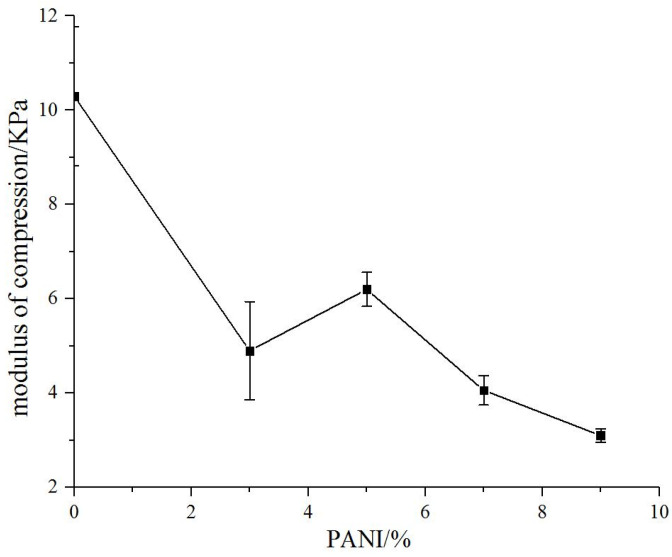
Compression modulus curve of the bio conductive materials with different polyaniline concentrations.

The compressive modulus of natural biomaterial substrate without polyaniline was 10.3 kPa. The compressive modulus of the conductive biomaterials decreased with the addition of polyaniline significantly (3.1–6.2 kpa) and gradually decreased with the increase in polyaniline concentration (Figure 4).

### 3.2. Electrical Properties

**Figure 5:**
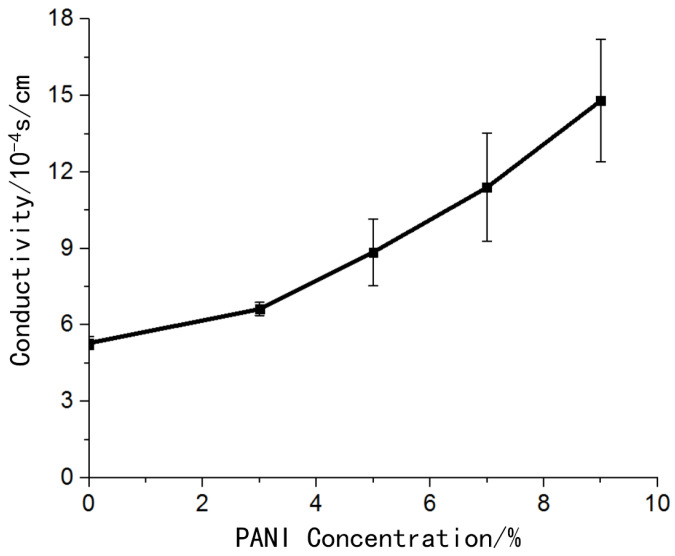
Conductivity change curve of bio-conductive materials with different polyaniline concentrations.

The hydrogel material without polyaniline also had certain conductivity. The addition of conductive polymer polyaniline improved the conductivity of the material, and the conductivity of the material increased as the concentration of polyaniline increases (Figure 5).

**Figure 6:**
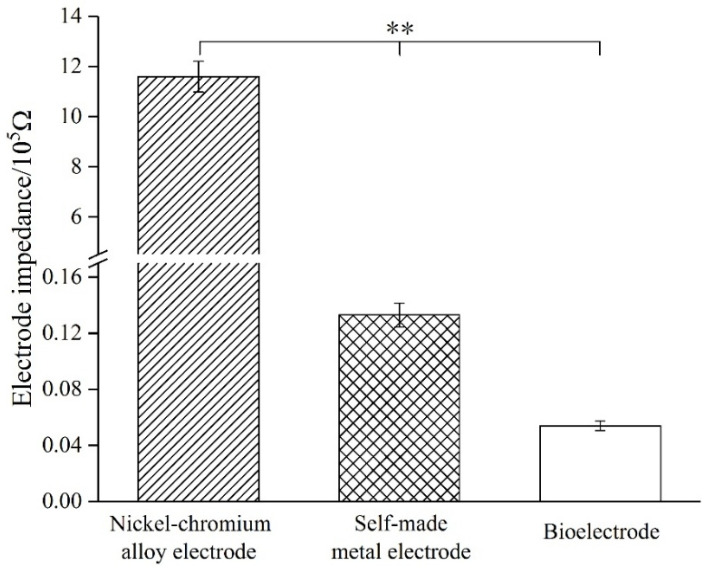
Standard nickel-chromium alloy electrode, self-made metal electrode and self-made bioelectrode impedance at 1 KHz (*n* = 4). (** *p* < 0.01).

In the vicinity of 1 KHz, the standard metal electrode impedance was 1.16 MΩ, while the self-made metal electrode impedance was 13.3 KΩ. After modification of the biological conductive material, the measured electrode impedance was 5.4 KΩ (Figure 6).

### 3.3. Biocompatibility In Vitro

**Figure 7:**
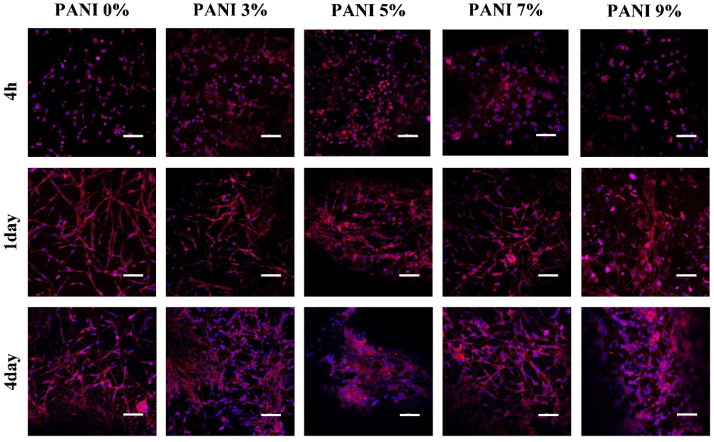
Surface morphology of U87 cells on bio-conductive materials with different concentrations of polyaniline (The white bar represents 100 μm).

The morphology of the cells was observed after being cultured for 4 h, 1 day and 4 days. After 4 h (Figure 7), the cells in each group were evenly attached to the surface of the material, and the cell bodies were ellipsoidal without spreading. At 1 day, the cells in each group spread out, and the cell bodies were spindle-shaped and connected with each other. The cells in the groups without polyaniline and 3% polyaniline were completely spread out, while the cells in other groups were partially spread out; At 4 days, the number of cells in the group without polyaniline and 3% polyaniline increased, and the network formed by cell spreading was denser, while in other groups, although the cells proliferated, the spreading phenomenon was not obvious.

### 3.4. Biocompatibility In Vivo

**Figure 8:**
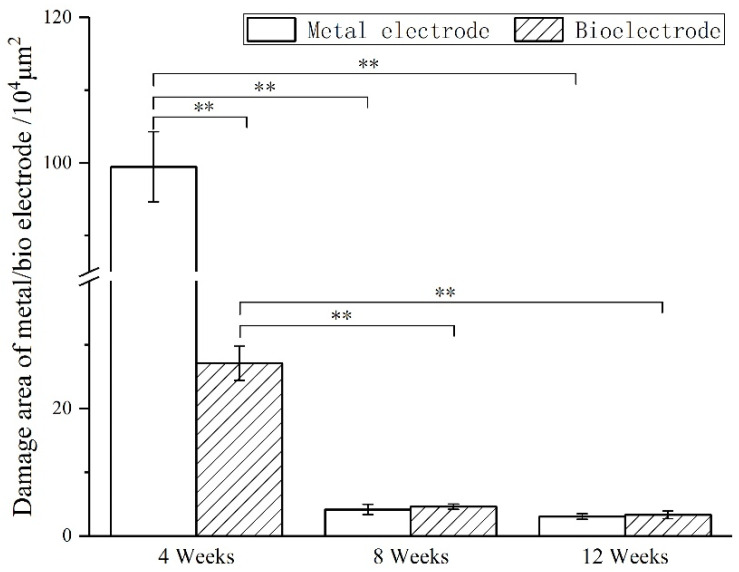
Damage area of metal/bio electrodes at different times.(** *p* < 0.01).

**Figure 9:**
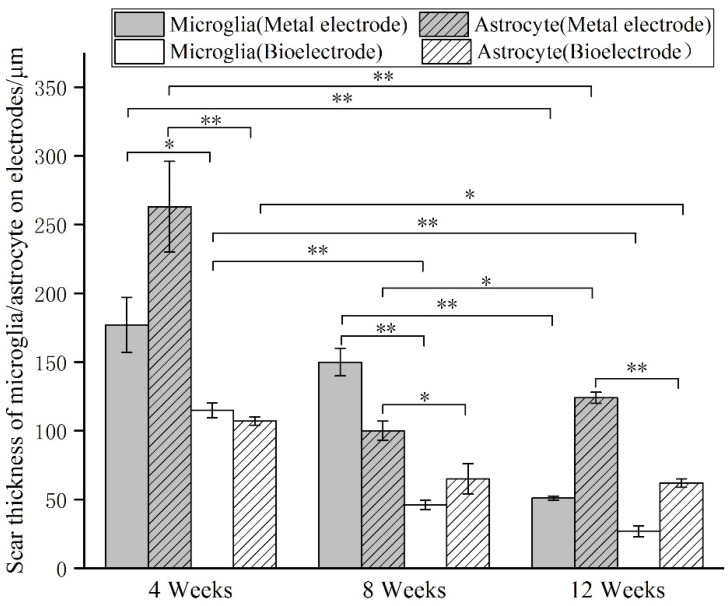
Scar thickness of microglia/astrocytes on metal/bio electrodes at different times. (* *p* < 0.05, ** *p* < 0.01).

The images of immunofluorescence staining were processed by MATLAB software to measure the area of brain defect and the thickness of the glial scar (Figure 8). The thickness of the glial scar was the average thickness of eight uniform positions on the scarring (Figure 9).

**Figure 10:**
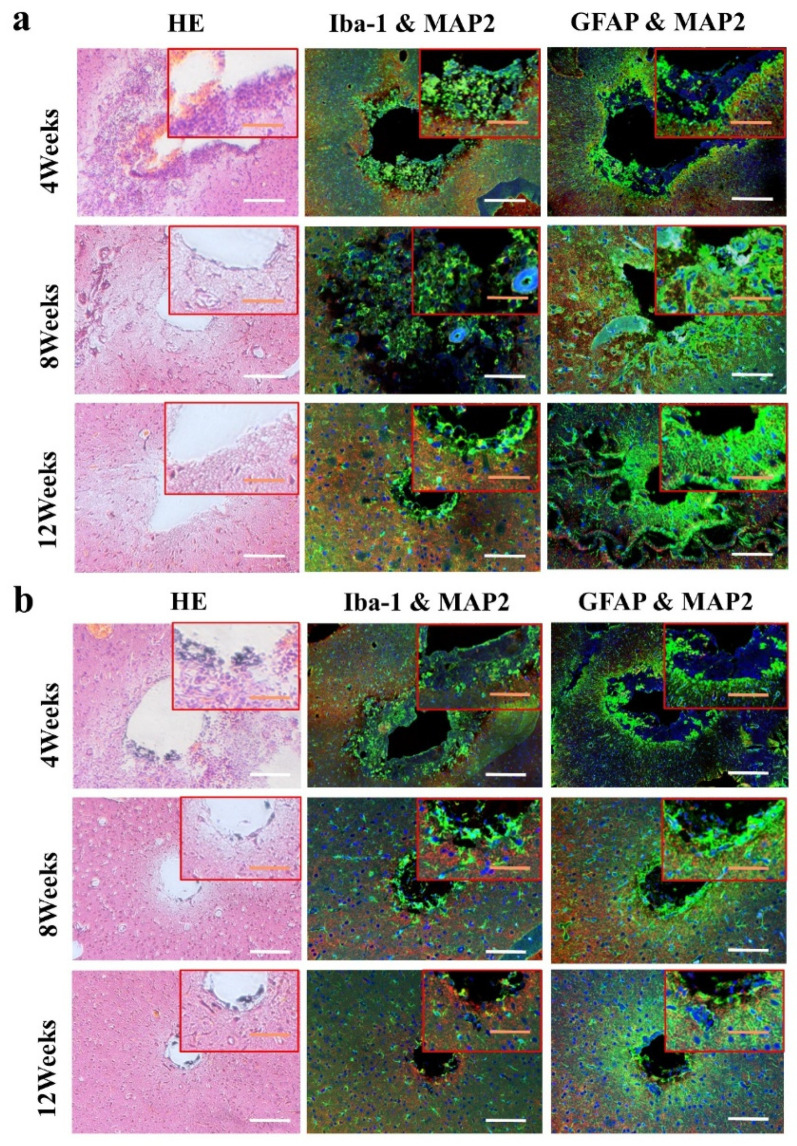
Immunohistochemical detection of electrode in vivo. (**a**) Metal electrode group; (**b**) bioelectrode group (Green: GFAP and Iba-1, astrocytes and microglial; red: MAP2, neuron; blue: DAPI, nucleus. The white bar represent 100 μm; the orange bar represent 50 μm).

At 4 weeks, the brain tissue was in the primary stage of the immune response. Besides the inflammatory cells, a large number of microglia were found around the electrodes. The astrocytes around the metal electrode were more than those around the biological electrode. At 8 weeks, there were still a large number of microglia around the metal electrode, while the number of microglia around the biological electrode decreased. At the same time, more astrocytes gathered around the two electrodes, and the number of astrocytes gathered around the metal electrode was more than that around the biological electrode. At 12 weeks, the number of microglia around the metal electrode decreased, but the astrocytes still existed in large quantities. However, the microglia around the biological electrode had basically disappeared, astrocytes also decreased, and the neuronal inclusions moved closer to the electrode (Figure 10).

### 3.5. Acute Electrophysiological Detection

**Figure 11:**
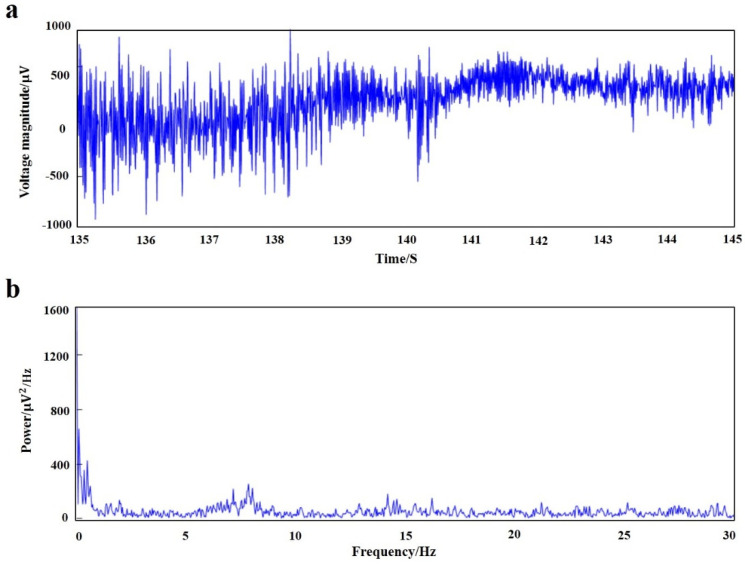
Acute electroencephalogram of anesthetized rats. (**a**) Local field potential time-domain waveform; (**b**) power spectral density.

MATLAB software was used to process the original signal, and the time domain and frequency domain waveforms of the EEG signals of rats under anesthesia were obtained (Figure 11). The fluctuation of local field potential can be clearly seen from the time domain diagram, and power spectral density showed that the EEG signals of acute detection were mainly concentrated in the low-frequency bands of <4 Hz and 6–8 Hz (δ wave and β wave).

## 4. Discussion

The addition of polyaniline significantly reduced the compressive modulus of the conductive biomaterials. The compressive modulus decreased with the increase in polyaniline concentration. When the polyaniline concentration was 9%, the compressive modulus of the material was reduced by 70%. Although the modulus of the material was greater than the natural brain tissue, it is much smaller than metal electrodes, which greatly protected the brain tissue at the implantation site. Although smaller compressive modulus would benefit the induction of nerve cells, it would also increase the difficulty of electrode manufacturing. Therefore, it was necessary to consider both the biological and mechanical properties to select a suitable polyaniline concentration material in the electrode design and preparation process.

Without the addition of conductive polymer, the pore size of the material was about 175 μm, and the pores of the material with polyaniline were less than 100 μm, and the pores decreased as the concentration of polyaniline increased. It could be seen from the 500 SEM images that the surface roughness of the micropores of the material increased with the increase in the polyaniline concentration, which was beneficial to increase the specific surface area of the material and enhanced the conductivity of the electrode. Excessive aggregation of polyaniline particles affected the growth of nerve cells, so moderate concentration polyaniline materials should be chosen.

The hydrogel material without polyaniline was also conductive because the ionic components in the hydrogel played a conductive role. The addition of polyaniline improved the conductivity of the material. The conductivity of the conductive material with a concentration of 9% polyaniline was 2.8 times that of the hydrogel material. The electrical conductivity of biological conductive materials was in the order of 10^−3^ S/cm, which met the requirements of electrode preparation for material conductivity.

Generally speaking, the signal amplitude of nerve cells is 50–1000 μV, and the frequency is about 1 kHz, so the impedance value of the electrode at 1 kHz is used as an important indicator to study the electrical conductivity of the electrode. The standard metal electrode impedance was 1.16 MΩ at 1 kHz, while the self-made metal electrode impedance was 13.3 KΩ. With the modification of the electrode bio-conductive material, the electrode impedance was reduced to 5.4 KΩ, which is 1/200 of the impedance of the standard metal electrode.

No matter whether polyaniline was added or not, U87 cells could be attached and spread on the surface of the material. Cell proliferation and spreading at a concentration of 3% polyaniline were similar to those on hydrogel materials. As the concentration of polyaniline increases, the cell proliferation ability increased, but the spreading ability was affected. This experiment showed that bio-conductive materials had good in vitro biocompatibility and the biological basis for preparing electrode materials.

At 4 weeks, the brain tissue was in the primary stage of the immune response. In addition to inflammatory cells, there were a large number of microglia around the electrodes. Microglia induced astrocytes to proliferate and gather around the electrodes. More astrocytes gathered around the metal electrode, indicating that the metal electrode produced a stronger immune response. At 8 weeks, there were still a large number of microglia around the metal electrodes, while the number of microglia around the biological electrode decreased. The number of astrocytes around the metal electrode was more than that around the biological electrode. At 12 weeks, the microglia around the metal electrode decreased, but astrocytes still existed in large numbers, indicated that the glial scars had formed around the metal electrode. The microglia and astrocytes around the bioelectrodes basically disappeared, and the neurons moved closer to the electrodes.

Although some inflammatory cells and microglia gathered around the bioelectrode at the initial stage of the immune response, these tissue reactions disappeared completely during the recovery process. The glial cells around the metal electrode continued to increase after the tissue inflammation and finally formed the glial scar. The results showed that the biological brain electrodes prepared in this research would not cause glial cell aggregation during long-term use, avoid the generation of glial scars and thus ensure the smoothness of the connection between the electrodes and nerve signals.

The results showed that the signals are mainly concentrated in <4 Hz and 6–8 Hz parts. The delta wave and theta wave account for the main components of the EEG signal in the rat under anesthesia. The biological brain electrodes could accurately record the brain’s local field potential signals.

## 5. Conclusions

A new type of electroprobe made in biocompatible natural materials was developed in this study, aiming to promote the communications and bonds between the neurons and the interface. Here, conductive-biomaterials-based interfaces were designed, and the mechanical and electrochemical properties, cytocompatibility with glioma cells and the inflammatory response were fully explored. Results indicated that compared with the metal electrode, the developed bio-electroprobe (3.1–6.2 KPa) has an elastic modulus closer to that of the natural brain tissue, and the electrochemical impedance of the bio-electrode (5.4 KΩ) was reduced by 200 times at 1 KHz. Cell-culture studies show that the composite biomaterials for the electrode were biologically friendly by forming complex and interconnected networks in 4-day culture. Moreover, the developed bio-probe was implanted in mouse brains for 12 weeks using a metal probe as control, and the histological study results indicated that the bio-probe could significantly reduce the glial encapsulation and neuron loss around the implants, which proved that the developed bio-electrode has the viability and potential to encourage neuron to bond in vivo. In conclusion, the conducting polymer/biomaterials probes may help improve the long-term performance of the neural electrodes with better biocompatibility and more efficient signal transmission.

## Data Availability

No new data were created or analyzed in this study. Data sharing is not applicable to this article.
